# *ALKBH1* rs2267755 C>T polymorphism decreases neuroblastoma risk in Chinese children

**DOI:** 10.7150/jca.89271

**Published:** 2024-01-01

**Authors:** Xinxin Zhang, Chunlei Zhou, Yemu Zhao, Changmi Deng, Haiyan Wu, Zhenjian Zhuo, Jing He

**Affiliations:** 1 Department of Pediatric Surgery, Guangzhou Institute of Pediatrics, Guangdong Provincial Key Laboratory of Research in Structural Birth Defect Disease, Guangzhou Women and Children's Medical Center, Guangzhou Medical University, Guangzhou 510623, Guangdong, China.; 2 Department of Pathology, Children's Hospital of Nanjing Medical University, Nanjing 210008, Jiangsu, China.; 3 Department of Pathology, Guangzhou Women and Children's Medical Center, Guangzhou Medical University, Guangzhou 510623, Guangdong, China.; 4 Laboratory Animal Center, School of Chemical Biology and Biotechnology, Peking University Shenzhen Graduate School, Shenzhen 518055, Guangdong, China.

**Keywords:** neuroblastoma, *ALKBH1*, polymorphism, susceptibility

## Abstract

Neuroblastoma is a highly malignant extracranial solid tumor in pediatrics. ALKBH1 as a recently discovered DNA N6-methyldeoxyadenosine (6mA) demethylase closely links to tumorigenesis. Whether the *ALKBH1* polymorphism contributes to neuroblastoma risk remains unclear. In the present study, we genotyped the *ALKBH1* single nucleotide polymorphisms (SNPs) in 402 neuroblastoma patients and 473 healthy controls by TaqMan assay. Odds ratios (ORs) and 95% confidence intervals (CIs) were also calculated to evaluate the strength of the association. Our result exhibited that the rs2267755 C>T (CT vs. CC, adjusted OR=0.69, 95% CI=0.50-0.94, *P*=0.019) is significantly associated with reduced neuroblastoma risk. And its protective effect is particularly significant in children with tumors originating from the retroperitoneal. Combined genotype analysis revealed that carriers with 1-2 protective genotypes are more susceptible to neuroblastoma than those with 3-4 protective genotypes (adjusted OR=0.71, 95% CI=0.53-0.97, *P*=0.028). Moreover, the rs2267755 C>T is significantly associated with messenger RNA (mRNA) expression of *ALKBH1* and three of its surrounding genes, including *SNWQ, ADCK1*, and *RPL21P10*. These results suggest that the rs2267755 C>T may be a genetic variant to reduce neuroblastoma risk.

## Introduction

Neuroblastoma is the most frequently extracranial solid tumor in pediatrics, accounting for nearly 15 % of all pediatric cancer deaths 1, 2. Although previous studies have identified partial genetics aberrations as potential neuroblastoma therapeutic targets, it is necessary to explore new regulators for developing new neuroblastoma therapies 2. In recent years, methylation has been recognized as playing an important role in neuroblastoma progression 3, 4. Multiple studies demonstrated that suppressing DNA m5C affects neuroblastoma cell proliferation and colony-forming activity 5, 6. Targeted inhibition of histone H3K27 demethylation effectively treats high-risk neuroblastoma. Moreover, RNA N6-methyladenosine methylation gene variations are significantly associated with neuroblastoma risk 8-12.

DNA N6-methyladenine (6mA) is a recently identified modification in mammals, which is widely present in the human genome 13. Like most methylation modifications, 6mA is an invertible DNA modification. The identification of its regulators had achieved definite progress. Studies demonstrated that the METTL4 could catalyze 6mA modification on genomic DNA (gDNA) and be active against mitochondria DNA *in vitro* 14, 15. ALKBH1 and ALKBH4 are responsible for DNA 6mA demethylation in mammals 16, 17.

Recently, more evidence suggested that 6mA is closely related to tumorigenesis 18, 19. Thereinto, silencing of *ALKBH1* significantly impaired the growth of cancer xenografts *in vivo*. Quyang L et al. proposed that *ALKBH1* is a potential therapeutic target to reduce the burden of vascular calcification. Mechanistically, they found that *ALKBH1* activates bone morphogenetic protein 2(*BMP2*) and aggravates osteogenic reprogramming in chronic kidney disease 20. A recent study revealed that *ALKBH1* is highly expressed in human head and neck squamous cell carcinoma cells and patient tissues, promoting cell proliferation by enhancing *DDX18* expression 21. More importantly, Rashad, S. et al uncovered that *ALKBH1* assists tRNA cleavage in demethylation stress in rat neuroblastoma cells 22. However, it remains unclear whether *ALKBH1* is associated with neuroblastoma risk.

The study of genetic variants is one of the most important parts to elucidate the role of key genes in neuroblastoma. Our previous studies clarified that SNPs of multiple genes are tightly related to neuroblastoma risk 23, 24. Given the crucial role of *ALKBH1* in cancer, we conducted a comprehensive analysis to illuminate the relationship between the *ALKBH1* gene SNPs and neuroblastoma susceptibility in Chinese children, providing new evidence for predicting the risk of neuroblastoma.

## Subjects and Methods

### Subjects

In this study, 402 neuroblastoma patients and 473 control samples were recruited from Children's Hospital of Nanjing Medical University in Jiangsu province, China (**Table S1**) 25, 26. All neuroblastoma patients were confirmed by histopathology or cytology, and did not receive radiotherapy and/or chemotherapy prior to blood collection. Patients with a history of malignancy in other organs were excluded. Control samples without neuroblastoma were recruited in the same hospital. All subjects' guardians had written informed consent at the start of this research. The present study was approved by the institutional review board of Children's Hospital of Nanjing Medical University (Approval No: 202112141-1).

### DNA extraction and selection of *ALKBH1* polymorphism

Total genomic DNA was extracted using the TIANamp Blood DNA Kit (TianGen Biotech Co., Ltd., Beijing, China) from EDTA-peripheral blood. As previously, *ALKBH1* potentially functional SNPs were chosen from the NCBI dbSNP database (https://www.ncbi.nlm.nih.gov) and SNPinfo (https://manticore.niehs.nih.gov), we selected these SNPs according to the following criteria: 1) located at both sides of *ALKBH1* (5'UTR, 5' near gene, 3'UTR or 3' near gene); 2) potential biological functions predicted by snpinfo; 3) the minor allele frequency in Chinese subject is >5%; 4) SNPs showing significant linkage disequilibrium (LD) with each other (R^2^≥0.8) are excluded. Consequently, we selected the four *ALKBH1* potential functional SNPs (rs2267755 C>T, rs1048147 C>A, rs6494 T>A, rs176942 A>G) with R^2^ lower than 0.5. The Taqman real-time PCR method was used to genotype the four *ALKBH1* SNPs under the blind status of neuroblastoma cases and control 27. To guarantee the reliability of the genotyping of *ALKBH1* SNPs, 10% of samples were randomly redetected and the accuracy was 100%.

### Statistics analysis

In this study, statistical analysis was conducted with the Chi-square test to evaluate the significant difference for categorical variables, and a *t*-test was employed for continuous variables. *ALKHB1* SNPs were identified with χ^2^ test to compare the difference between the cases and control samples, which agreed with Hardy-Weinberg equilibrium (HWE). To investigate the association between each *ALKBH1* SNP and neuroblastoma risk, odds ratios (ORs) with 95% confidence intervals (CIs) were calculated using unconditional logistic regression. This statistical analysis was also performed based on age, gender, and clinical stages. For all statistical tests on two sides, *P*-value < 0.05 was considered statistically significant, which was calculated by SAS software (Version 10.0, SAS Institute, Inc., Cary, North Carolina).

## Result

### Association of *ALKBH1* gene polymorphism with neuroblastoma susceptibility

In this study, genotyping of *ALKBH1* was performed in 402 neuroblastoma patients and 473 controls, whose clinical characteristics were listed in **Table S1**. As shown in **Table 1**, we successfully genotyped four *ALKBH1* SNPs (rs2267755 C>T, rs1048147 C>A, rs6494 T>A, rs176942 A>G) in 399 cases and 473 controls samples, which coincided with Hardy-Weinberg equilibrium (HWE) among controls (HWE *P* > 0.05). Thereinto, the rs2267755C>T variant was significantly associated with neuroblastoma risk (CT versus CC: adjusted OR=0.69, 95% CI=0.50-0.94, *P*=0.019; CT/TT versus CC: adjusted OR=0.74, 95% CI=0.55-0.98, *P*=0.039). We then identified rs2267755 CT/TT, rs1048147 CC, rs6494 TA/TT, and rs176942 AG/AA) as protective genotypes following their values of ORs. The combined genotype result showed that individuals with 3-4 protective genotypes experienced a 0.71-fold decrease in the risk of development neuroblastoma when compared with those with 1-2 protective genotypes (95% CI=0.53-0.97, *P*=0.028).

### Stratification analysis

We next evaluated the correlation between rs2267755 C>T variant with neuroblastoma susceptibility in subgroups divided by age, gender, sites of origins, and INSS stages (**Table 2**). Stratified analysis results revealed that the rs2267755 C>T was associated with reduced neuroblastoma risk in children with tumors originating from the retroperitoneal (adjusted OR=0.60, 95% CI=0.41-0.87, *P*=0.007). Besides, individuals with 3-4 protective genotypes significantly decreased neuroblastoma susceptibility compared with those with 1-2 protective genotypes in a subgroup of tumors originating from retroperitoneal (adjusted OR=0.64, 95% CI=0.43-0.95, *P*=0.026).

### Effect of rs2267755C>T on the expression of ALKBH1

**Figure 1:**
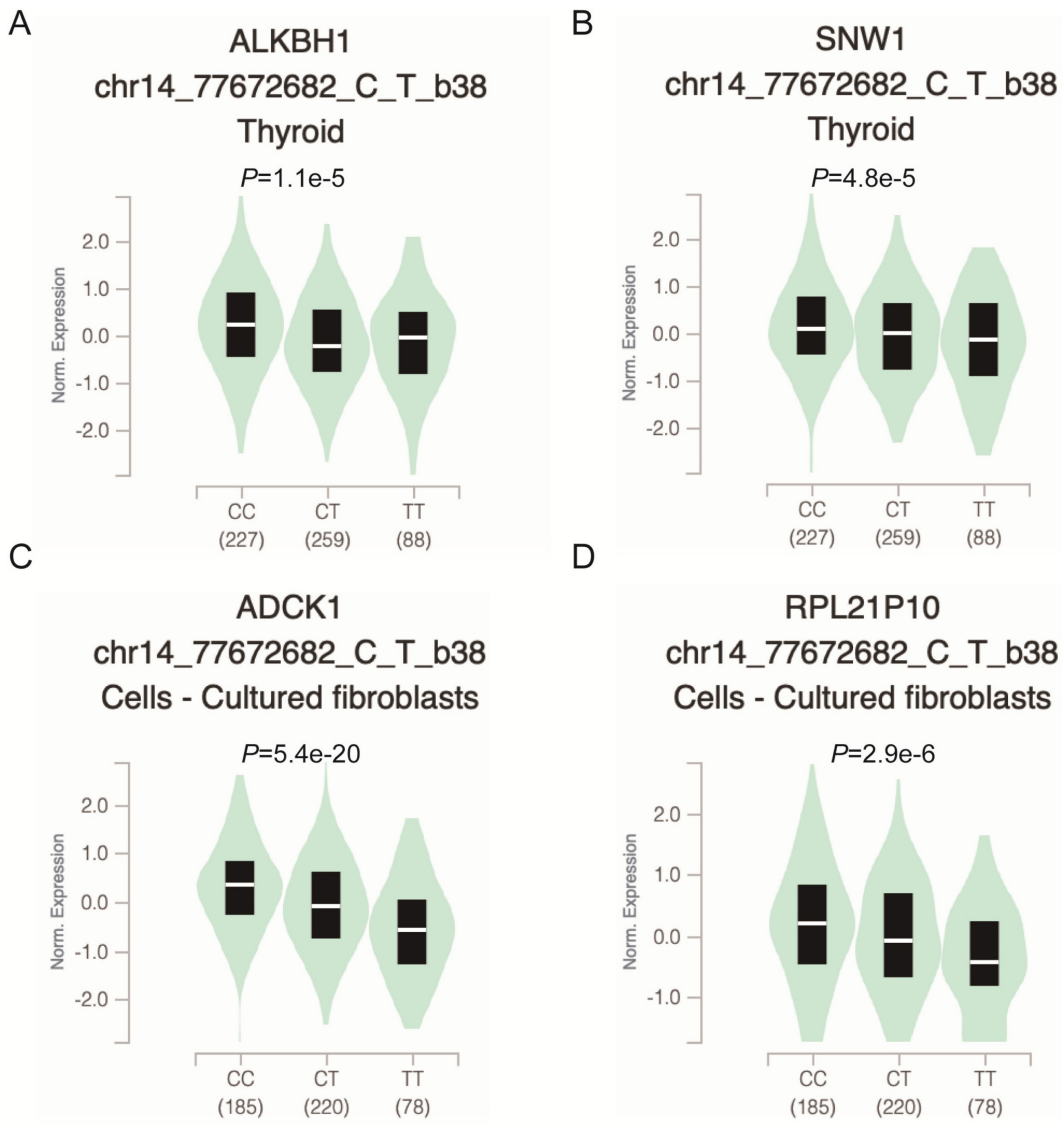
Functional relevance of rs2267755 C>T on gene expression in GTEx Database. rs2267755 C>T significantly reduced *ALKBH1* (A) and *SNW1* (B) mRNA expression in the thyroid (*P*=1.1e-5, *P*=4.8e-5) as well as in *ADCK1* (C) and *RPL21P10* (D) in the cell-cultured fibroblasts (*P*=5.4e-20, *P*=2.9e-6).

To further explore whether the functional relevance of rs2267755C>T affects the messenger (mRNA) expression of adjacent genes, we used the GTEx (https://www.gtexportal.org/) portal to investigate Cis-expression quantitative trait loci (eQTL) target genes of the rs2267755C>T. Result manifested that the rs2267755 T allele was significantly associated with reduced mRNA expression of *ALKBH1* and *SNW1* in the thyroid (**Figure 1A, B**), as well as *ADCK1* and *RPL21P10* in the cultured fibroblasts cell (**Figure 1C, D**).

## Discussion

Understanding the role of 6mA modification will provide new insight into the prediction of neuroblastoma and the establishment of new treatment strategies. This study investigated the relationship between 6mA demethylase *ALKBH1* and neuroblastoma susceptibility in Chinese children from the perspective of genetic polymorphism. Our research results showed that the *ALKBH1* rs2267755C>T variant is significantly associated with reduced neuroblastoma risk. More protective genotypes significantly decreased neuroblastoma susceptibility in Chinese children.

DNA 6mA modification is recently identified in mammals 28. Its balance is synergistically regulated by methyltransferases (METTL4) and demethylase (ALKBH1, ALKBH4). Methylation-related studies demonstrated that genetic variants of the methylation-related genes could affect tumor susceptibility 12, 29, 30. Our group previously genotyped 5 N6-methyladenosine (m6A) methylase *METTL14* SNPs in 898 neuroblastoma patients and 1734 control samples. We found that 3 SNPs are significantly associated with neuroblastoma risk 8. Besides, we also found a significant association between m6A methylase *METTL3* gene SNP rs1061027 C>A variant and neuroblastoma susceptibility in females and ≤ 18-month children 9. These results suggested that variation of methylation gene is associated with neuroblastoma risk. However, it is necessary to explore the correlation between 6mA regulator SNPs and neuroblastoma risk.

Recent studies verified that ALKBH1 as DNA 6mA demethylase plays a vital role in human cancer 31, 32. At present, the function mechanism of ALKBH1 is still at the initial stage. Consistent with FTO and ALKBH5, ALKBH1 is a member of the AlkB family, which catalyzes substrate demethylation in Fe (II)- and α-ketoglutarate-dependent manner 32. Previous research found that the *FTO* (*ALKBH9*) gene SNP rs8047395 A>G variant is significantly correlated with Wilms tumor 29. And the *ALKBH5* gene SNPs had a weak impact on hepatoblastoma risk 33. Therefore, we predicted ALKBH1 may be associated with neuroblastoma risk. In the present study, we explored the role of the *ALKBH1* SNPs in neuroblastoma risk for the first time. Among 4 newly identified *ALKBH1* SNPs, the rs2267755 C>T is significantly associated with neuroblastoma risk in Chinese children. Moreover, the protective effect of rs2267755 C>T is significantly increased in children with tumors originating from retroperitoneal.

**Figure 2:**
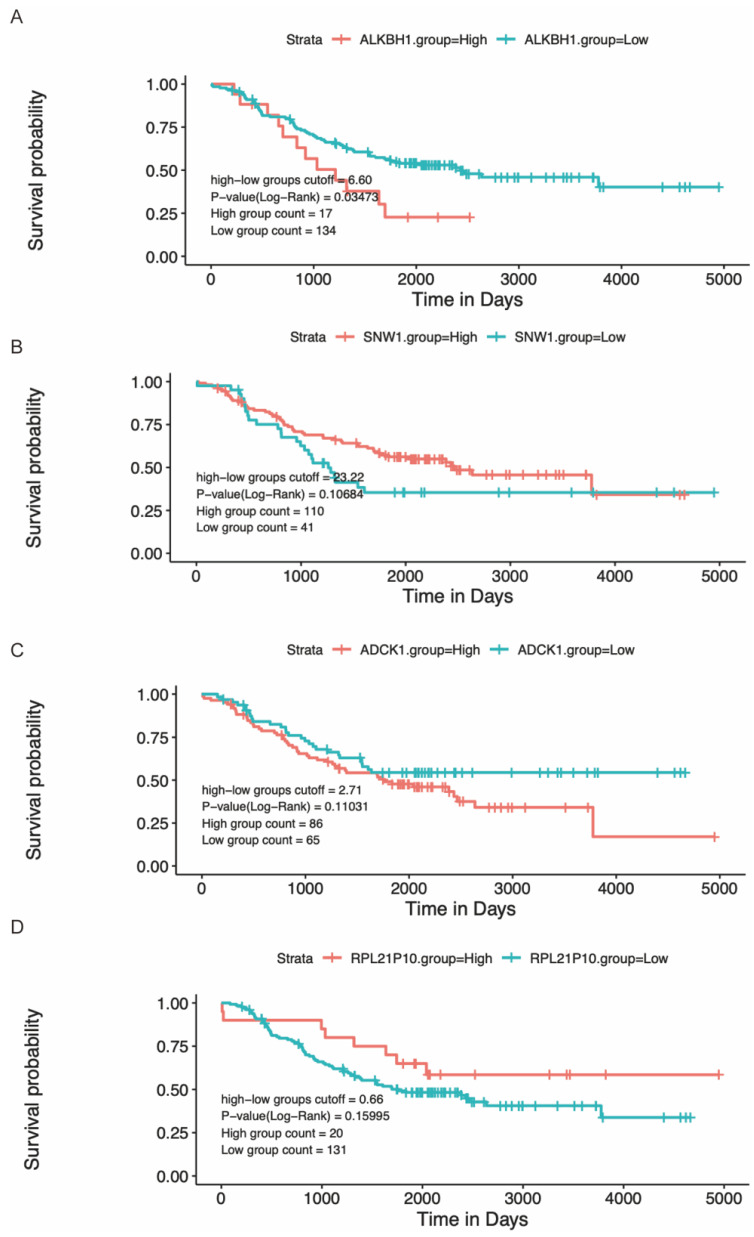
The correlation between gene levels and survival probability of neuroblastoma patients.

Modulating gene expression is one of the most important ways for regulators to affect tumor development. Consistent with this, lacking ALKBH1 affected the expression of developmentally important miRNAs in embryonic stem cells 36. Depletion of ALKBH1 inhibits the expression of adipogenic-related genes *CEBPA*, *PPARG*, *PLIN1*, and *ADIPOQ* in human mesenchymal stem cells 37. Therefore, we evaluated the effect of rs2267755 C on ALKBH1 and surrounding gene expression. Harnessing the GTEx database (Figure 1), we found that rs2267755 C is significantly correlated with the expression of *ALKBH1* in the thyroid, surrounding gene *SNW1* in the thyroid, and *ADCK1* and *RPL21P10* in the cultured fibroblasts cells. Moreover, we explore the survival probability of neuroblastoma patients among these relevant genes through the PCAT (PDX for childhood cancer Therapeutics http://pedtranscriptome.org/?home), in which the statistical analysis (Figure 2) shows that ALKBH1 level is negatively correlated with the survival probability of neuroblastoma patients. The other three gene expression levels are not significant. Therefore, we speculate rs2267755 C>T is associated with increased survival rates of neuroblastoma patients. More experiments are needed to confirm the role of rs2267755C in modulating gene expression.

In general, we elucidated the relationship between *ALKBH1* gene SNPs and neuroblastoma susceptibility. Our study revealed a significant correlation between rs2267755C>T variant and neuroblastoma risk in Chinese children. Further studies were needed to illustrate the biological mechanisms.

## Supplementary Material

Supplementary table.

## Figures and Tables

**Table 1:** *ALKBH1* gene polymorphisms and neuroblastoma susceptibility in Jiangsu children.

Genotype	Cases (N=399)	Controls (N=473)	*P* ^ a^	Crude OR (95% CI)	*P*	Adjusted OR (95% CI) ^b^	*P* ^ b^
rs2267755 C>T (HWE=0.262)							
CC	131 (32.83)	125(26.43)		1.00		1.00	
CT	179 (44.86)	248 (52.43)		**0.69 (0.50-0.94)**	**0.019**	**0.69 (0.50-0.94)**	**0.019**
TT	89 (22.31)	100 (21.14)		0.85 (0.58-1.24)	0.395	0.85 (0.58-1.24)	0.397
Additive			0.278	0.90 (0.75-1.09)	0.278	0.90 (0.75-1.09)	0.279
Dominant	268 (67.17)	348 (73.57)	0.039	**0.74 (0.55-0.98)**	**0.039**	**0.74 (0.55-0.98)**	**0.039**
Recessive	310 (77.69)	373 (78.86)	0.678	1.07 (0.78-1.48)	0.677	1.07 (0.78-1.48)	0.673
rs1048147 C>A (HWE=0.266)							
CC	197 (49.37)	250 (52.85)		1.00		1.00	
CA	164 (41.10)	181 (38.27)		1.15 (0.87-1.52)	0.332	1.15 (0.87-1.52)	0.335
AA	38 (9.52)	42 (8.88)		1.15 (0.71-1.85)	0.570	1.15 (0.71-1.85)	0.572
Additive			0.354	1.10 (0.90-1.35)	0.353	1.10 (0.90-1.35)	0.356
Dominant	202 (50.63)	223 (47.15)	0.306	1.15 (0.88-1.50)	0.306	1.15 (0.88-1.50)	0.309
Recessive	361 (90.48)	431 (91.12)	0.743	1.08 (0.68-1.71)	0.742	1.08 (0.68-1.71)	0.742
rs6494 T>A (HWE=0.974)							
TT	349 (87.47)	400 (84.57)		1.00		1.00	
TA	45 (11.28)	70 (14.80)		0.74 (0.49-1.10)	0.136	0.74 (0.49-1.10)	0.133
AA	5 (1.25)	3 (0.63)		1.91 (0.45-8.05)	0.378	1.94 (0.46-8.19)	0.369
Additive			0.380	0.85 (0.60-1.22)	0.380	0.85 (0.60-1.21)	0.380
Dominant	50 (12.53)	73 (15.43)	0.220	0.79 (0.53-1.16)	0.221	0.78 (0.53-1.16)	0.219
Recessive	394 (98.75)	470 (99.37)	0.340	1.99 (0.47-8.37)	0.349	2.01 (0.47-8.49)	0.342
rs176942 A>G (HWE=0.788)							
AA	276 (69.17)	334 (70.61)		1.00		1.00	
AG	102 (25.56)	126 (26.64)		0.98 (0.72-1.33)	0.895	0.98 (0.72-1.33)	0.896
GG	21 (5.26)	13 (2.75)		1.96 (0.96-3.98)	0.064	1.97 (0.97-4.01)	0.062
Additive			0.290	1.14 (0.90-1.45)	0.290	1.14 (0.90-1.45)	0.288
Dominant	123 (30.83)	139 (29.39)	0.644	1.07 (0.80-1.43)	0.644	1.07 (0.80-1.43)	0.642
Recessive	378 (94.74)	460 (97.25)	0.056	1.97 (0.97-3.98)	0.060	1.98 (0.98-4.01)	0.059
Combine protective genotypes ^c^							
0-2	119 (29.82)	110 (23.26)		1.00		1.00	
3-4	280 (70.18)	363 (76.74)	0.028	**0.71 (0.53-0.97)**	**0.028**	**0.71 (0.53-0.97)**	**0.029**

OR, odds ratio; CI, confidence interval, HWE, Hardy**-**Weinberg equilibrium. ^a^ χ^2^ test for genotype distributions between neuroblastoma patients and cancer-free controls. ^b^ Adjusted for age and sex. ^c^ Protective genotypes were carriers with rs2267755 CT/TT, rs1048147 CC, rs6494 TA/TT, and rs176942 AG/AA genotypes.

**Table 2:** Stratification analysis for the association between *ALKBH1* gene protective genotypes and neuroblastoma risk in Jiangsu children.

Variables	rs2267755 (cases/controls)		AOR (95% CI) ^a^	*P* ^a^	Protective genotypes (cases/controls)		AOR (95% CI) ^a^	*P* ^a^
	CC	CT/TT			0-2	3-4		
Age, month								
≤18	46/33	90/106	0.61 (0.36-1.03)	0.065	40/28	96/111	0.61 (0.35-1.06)	0.076
>18	85/92	178/242	0.80 (0.56-1.13)	0.205	79/82	184/252	0.76 (0.53-1.09)	0.135
Sex								
Females	67/62	124/163	0.70 (0.46-1.07)	0.099	59/56	132/169	0.74 (0.48-1.14)	0.174
Males	64/63	144/185	0.77 (0.51-1.16)	0.209	60/54	148/194	0.69 (0.45-1.05)	0.086
Sites of origin								
Adrenal gland	22/125	71/348	1.16 (0.69-1.95)	0.577	22/110	71/363	0.98 (0.58-1.66)	0.940
Retroperitoneal	62/125	103/348	**0.60 (0.41-0.87)**	**0.007**	53/110	112/363	**0.64 (0.43-0.95)**	**0.026**
Mediastinum	39/125	80/348	0.74 (0.48-1.14)	0.165	37/110	82/363	0.67 (0.43-1.05)	0.077
Others	7/125	11/348	0.56 (0.21-1.49)	0.248	6/110	12/363	0.60 (0.22-1.65)	0.324
INSS stages								
I+II+4s	54/125	119/348	0.79 (0.54-1.16)	0.231	51/110	122/363	0.73 (0.49-1.08)	0.116
III+IV	52/125	111/348	0.76 (0.52-1.13)	0.174	46/110	117/363	0.77 (0.51-1.15)	0.199

AOR, adjusted odds ratio; CI, confidence interval. ^a^Adjusted for age and sex, omitting the corresponding stratification factor.

## Abbreviations

6mA: DNA N6-methyldeoxyadenosine
SNP: single nucleotide polymorphism
m6A: RNA N6-methyldeoxyadenosine
mRNA: messenger RNA
HWE: Hardy-Weinberg equilibrium
AOR: adjusted odds ratio
CI: confidence interval
eQTL: Cis-expression quantitative trait loci
gDNA: genomic DNA
